# Identification of factors associated with good response to growth hormone therapy in children with short stature: results from the ANSWER Program^®^

**DOI:** 10.1186/1687-9856-2011-6

**Published:** 2011-07-07

**Authors:** Peter A Lee, John Germak, Robert Gut, Naum Khutoryansky, Judith Ross

**Affiliations:** 1 Department of Pediatrics, Milton S. Hershey Medical Center, Penn State College of Medicine, Hershey, PA, USA; 2 Novo Nordisk Inc, Princeton, NJ, USA; 3 Department of Pediatrics, Thomas Jefferson University duPont Hospital for Children, Philadelphia, PA, USA

## Abstract

**Objective:**

To identify factors associated with growth in children on growth hormone (GH) therapy using data from the American Norditropin Studies: Web-enabled Research (ANSWER) Program^® ^registry.

**Methods:**

GH-naïve children with GH deficiency, multiple pituitary hormone deficiency, idiopathic short stature, Turner syndrome, or a history of small for gestational age were eligible (N = 1,002). Using a longitudinal statistical approach, predictive factors were identified in patients with GHD for change from baseline in height standard deviation score (ΔHSDS) following 2 years of treatment.

**Results:**

Gradual increases in ΔHSDS over time were observed for all diagnostic categories. Significant predictive factors of ΔHSDS, ranked by significance were: height velocity (HV) at 4 months > baseline age > baseline HSDS > baseline body mass index (BMI) SDS > baseline insulin-like growth factor I (IGF-I) SDS; gender was not significant. HV at 4 months and baseline BMI SDS were positively correlated, whereas baseline age, HSDS, and IGF-I SDS were negatively correlated with ΔHSDS.

**Conclusions:**

These results may help guide GH therapy based on pretreatment characteristics and early growth response.

## Introduction

Treatment with exogenous growth hormone (GH) has become a well-accepted therapeutic option for children with growth failure. Since the availability of recombinant human GH (rhGH) in 1985, a wide range of conditions associated with decreased growth, including GH deficiency (GHD), Turner syndrome (TS), Noonan syndrome (NS), children born small for gestational age (SGA), Prader-Willi syndrome (PWS), idiopathic short stature (ISS), and SHOX (short stature homeobox) gene haploinsufficiency have been approved by the United States Food and Drug Administration (FDA) for treatment [1-4].

Treatment with GH has been demonstrated to increase both short-term growth and adult height in pediatric patients with a variety of different growth disorders [5-8]. However, considerable variability in response to this treatment has been reported across and within different diagnostic categories [9-11]. Such variability makes decisions about whether to treat with GH, when to begin treatment, and what dosing to use more difficult [12].

Reports from clinical trials and analyses suggest multiple factors that influence the response to GH treatment. Variables associated with better responses to GH treatment in patients with ISS include first-year growth response, younger age at start of treatment, the difference in height at the start of treatment from target height SD score (HSDS), and GH dose [13,14]. Additional factors may include underlying genetic conditions, presence of concomitant illness, and compliance with treatment [15].

Formal height prediction models have been developed that combine information regarding patient- and treatment-related factors. Such prediction models of response to GH have been developed for patients with isolated or idiopathic GHD [16-20], SGA [21-23], chronic kidney disease [24], ISS [25], and TS [26]. These models have the potential to aid individualized GH treatment planning and the adjustment of therapy based on early responses [27]. However, even though GH treatment regimens can be based on model-derived predictions of growth response [28], existing models account for only about one-half of the variability in the response to GH. Addition of genetic, biochemical, and other new variables to existing models may improve their accuracy and clinical utility [29,30].

Since 2002, the ANSWER (American Norditropin Studies: Web-enabled Research) Program^® ^registry has collected information on patients receiving Norditropin. Participation within the ANSWER Program is at the discretion of the participating physicians and includes diagnostic categories in which treatment with growth hormone is used. The aim of this paper is to report growth response among different diagnostic categories and to identify factors associated with greater growth response over the first 2 years in children with GHD undergoing treatment with GH.

## Methods

### Answer program registry

Data for this analysis were obtained from the ANSWER Program registry, a collection of long-term efficacy and safety information from patients treated with Norditropin^® ^(somatropin [rDNA origin] injection, Novo Nordisk A/S, Denmark) [31] in the United States. Patient histories and physical examination data were entered by participating physician investigators using the ANSWER Program registry reporting form, a web-based data entry tool. Informed consent was obtained in all cases. While the registry enrolls GH-treatment-naïve and non-naïve patients, for the purpose of this analysis, only naïve patient data from the following diagnostic categories were included in the current analyses: 1) GHD (isolated/idiopathic), 2) multiple pituitary hormone deficiency (MPHD), 3) TS, 4) SGA, and 5) ISS.

### Study description

Patient data collected at the first visit and/or follow-up visits included age, gender, GH dose, HSDS, insulin-like growth factor-I (IGF-I) SDS, body mass index (BMI) SDS, bone age, and annualized height velocity (HV). The maximal stimulated serum GH concentration was also recorded. Height and BMI SDS (z scores) were calculated according to the standard formulas provided by the Center for Disease Control and Prevention [32]. IGF-I SDS scores were calculated using a standard algorithm and reference values provided by Diagnostic Systems Laboratories, Inc. (Webster, TX, USA). Data were collected at baseline and at 4 months, 1 year, and 2 years of GH treatment. Data at 4 months were collected within a 1-month window and data at 1 and 2 years were collected within a 3-month window. To eliminate the potential of erroneous data having been entered, the following rules were used to remove patients from the analysis: lack of height information at baseline, 4 months, 1 year, or 2 years; baseline age 0 or > 18 years; baseline HSDS less than -5 or greater than +2; and baseline height < 35 cm or > 200 cm. Also, patients were excluded when key variables from baseline or subsequent values were deemed physically or chronologically implausible (3.77% of potential subjects were excluded according to this criteria).

### Regression model development

In this study, a longitudinal statistical approach was used to identify factors that have significant predictive value for change in HSDS from baseline (ΔHSDS) in a regression model. ΔHSDS data collected following the first and second years of treatment were included in the model. A smoothing procedure was applied for the corresponding mean value curves for first-year HV and baseline age. Due to the limited number of patients in the MPHD, TS, SGA, and ISS diagnostic categories, regression analysis was only performed for patients with GHD. The curves presented were built using polynomial regression. The quadratic polynomial regression, under the assumption that the height SD is not a function of baseline age, provided a sufficient fitting, while higher terms (for example, cubic and fourth degree) were not statistically significant.

## Results

### Baseline demographics

The ANSWER Program registry (as of November 30, 2009) contained information for over 9,000 patients, of which 1,002 GH treatment-naïve patients from selected diagnostic categories (GHD, MPHD, TS, SGA, and ISS) met the criteria for inclusion in this analysis. Baseline demographic characteristics for the subjects in this study by specific diagnostic category are summarized in Table 1. The study included longitudinal data for 698 patients with GHD, 71 with MPHD, 60 with TS, 50 with SGA, and 123 with ISS. Mean baseline ages were lower for MPHD (6.4 years), SGA (7.1 years), and TS (8.5 years) groups compared to those for GHD (10.9 years) or ISS (11.2 years). Baseline mean peak GH levels were lowest for patients with GHD and MPHD (5.5 and 3.6 ng/mL, respectively). Baseline mean GH dose (μg/kg/day) for the different diagnostic categories was the lowest for MPHD patients, consistent with their apparently greater degree of GH deficiency and associated GH sensitivity. For all diagnostic categories, the mean and median GH dose did not increase more than 0.007 mg/kg/day over the two years, indicating a very narrow GH dose change over this period.

**Table 1:** Baseline demographics by diagnostic category.

	GHD		MPHD		Turner		SGA		ISS	
	**n**	**Mean (SD)**	**n**	**Mean (SD)**	**n**	**Mean (SD)**	**n**	**Mean (SD)**	**n**	**Mean (SD)**

Gender										
Male	543	-	53	-	0	-	33	-	91	-
Female	155	-	18	-	60	-	17	-	32	-
Age	698	10.9 (3.46)	71	6.4 (5.23)	60	8.5 (4.17)	50	7.1 (3.41)	123	11.2 (2.88)
HSDS	698	-2.2 (0.86)	71	-2.0 (1.36)	60	-2.5 (0.77)	50	-2.8 (0.97)	123	-2.3 (0.68)
IGF-I SDS	605	-2.5 (1.26)	31	-3.2 (1.54)	34	-2.0 (1.43)	32	-2.1 (1.53)	114	-2.2 (1.11)
BMI SDS	681	-0.1 (1.38)	49	0.5 (1.84)	55	0.5 (0.97)	49	-0.8 (1.35)	123	-0.8 (3.38)
Bone Age, yrs	616	9.4 (3.31)	39	8.0 (4.58)	43	7.8 (3.45)	44	6.1 (3.36)	115	9.7 (2.93)
Peak GH, ng/mL	606	5.5 (2.69)	40	3.6 (3.03)	5	12.5 (8.25)	17	13.8 (10.95)	97	15.2 (8.10)
GH dose, μg/kg/day	697	45.9 (10.1)	71	40.6 (11.2)	60	51.9 (9.0)	50	49.9 (13.5)	123	46.1 (8.6)

BMI, body mass index; GH, growth hormone; GHD, growth hormone deficiency; HSDS, height standard deviation score; IGF-I, insulin-like growth factor I; ISS, idiopathic short stature; MPHD, multiple pituitary hormone deficiency; SD, standard deviation; SDS, standard deviation score; SGA, small for gestational age

### Height outcomes

**Figure 1:**
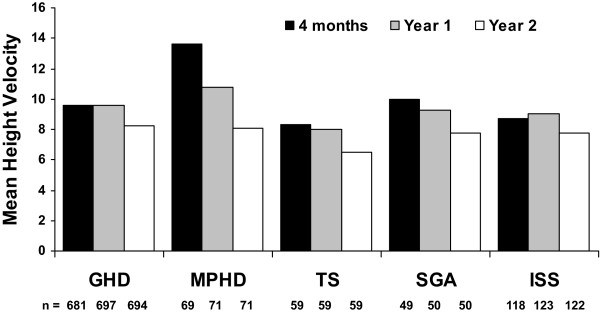
**Height velocity for all diagnostic categories over time**.

The effects of GH treatment on ΔHSDS over 2 years of treatment are shown in Table 2. Gradual increases in ΔHSDS were observed over time and ranged between 0.15 (ISS) to 0.37 (MPHD) at 4 months, and 0.82 (TS) to 1.20 (MPHD) at 2 years, with the largest ΔHSDS observed at year 1 and year 2 in patients with MPHD and SGA. Annualized HV at 4 months was 13.6 cm/year for MPHD, and between 8.33 (TS) and 9.96 (SGA) cm/year for the other indications (Figure 1). Within each diagnostic category, mean annualized HV was the greater during the first year, and generally decreased during the second year. Mean annualized HV at 1 year was greatest for MPHD at 10.74 cm/year, and ranged between 7.97 (TS) and 9.57 (GHD) for the other indications.

**Table 2:** HSDS and ΔHSDS by diagnostic category.

	HSDS		ΔHSDS	
	**n**	**Mean (SD)**	**n**	**Mean (SD)**

**GHD**				
Baseline	698	-2.22 (0.86)	-	-
4 Months	698	-2.03 (0.82)	698	0.19 (0.33)
Year 1	698	-1.61 (0.83)	698	0.61 (0.49)
Year 2	697	-1.17 (0.88)	697	1.06 (0.64)
**MPHD**				
Baseline	71	-1.98 (1.36)	-	-
4 Months	70	-1.62 (1.30)	70	0.37 (0.68)
Year 1	70	-1.13 (1.04)	70	0.85 (0.76)
Year 2	70	-0.79 (1.04)	70	1.20 (1.04)
**Turner**				
Baseline	60	-2.49 (0.77)	-	-
4 Months	59	-2.32 (0.82)	59	0.18 (0.20)
Year 1	60	-1.99 (0.86)	60	0.50 (0.31)
Year 2	60	-1.68 (0.90)	60	0.82 (0.43)
**SGA**				
Baseline	50	-2.76 (0.97)	-	-
4 Months	50	-2.48 (0.88)	50	0.28 (0.47)
Year 1	50	-1.96 (0.93)	50	0.80 (0.59)
Year 2	50	-1.59 (1.00)	50	1.18 (0.65)
**ISS**				
Baseline	123	-2.31 (0.68)	-	-
4 Months	123	-2.16 (0.69)	123	0.15 (0.19)
Year 1	123	-1.77 (0.69)	123	0.54 (0.38)
Year 2	123	-1.41 (0.79)	123	0.90 (0.59)

GHD, growth hormone deficiency; HSDS, height standard deviation score; ISS, idiopathic short stature; MPHD, multiple pituitary hormone deficiency; SD, standard deviation; SGA, small for gestational age.

### Regression analysis

Linear regression was performed on HSDS data for patients with GHD (Table 3). Variables significantly associated with ΔHSDS 1 and 2 years included HV at 4 months, and baseline age, HSDS, BMI SDS, and IGF-I SDS. The ranking of importance of predictive factors as related to ΔHSDS (as determined by the F value, the higher the more influential) was as follows: HV at 4 months > baseline age > baseline HSDS > baseline BMI SDS > baseline IGF-I SDS. HV at 4 months and baseline BMI SDS were positively correlated with ΔHSDS, while baseline age, HSDS, and IGF-I SDS were negatively correlated with ΔHSDS. Gender was less influential than the above factors (Table 3) and was not detected as statistically significant in this analysis.

**Table 3:** Regression model for longitudinal ΔHSDS at year 1 and year 2 for patients with GHD (n = 698).

Characteristic	β Estimate	F Value	*P *Value
Height velocity at 4 months	0.0319	214.31	< .0001
Baseline age	-0.0439	74.17	< .0001
Baseline HSDS	-0.0776	29.11	< .0001
Baseline BMI SDS	0.0398	20.62	< .0001
Baseline IGF-I SDS	-0.0245	5.74	.0169
Gender	-0.0438	2.46	.1175

BMI, body mass index; GHD, growth hormone deficiency; HSDS, height standard deviation score; IGF-I, insulin-like growth factor I; SDS, standard deviation score.

**Figure 2:**
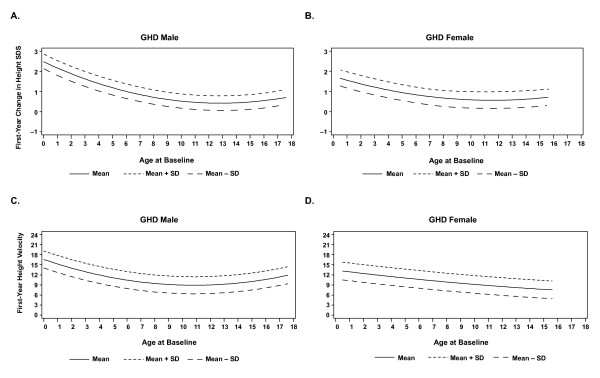
**First-year change from baseline HSDS and height velocity vs baseline age for patients with GHD (A, Change in baseline HSDS in male patients with GHD; B, Change in baseline HSDS in female patients with GHD; C, Change in height velocity in male patients with GHD; D, Change in height velocity in female patients with GHD)**.

Analysis of the mean values was used to build smoothed curves for demonstration of the relationship between first-year ΔHSDS and baseline age (Figure 2A and 2B), and between first-year HV and baseline age (Figure 2C and 2D) in male and female patients with GHD. Generally, the curves demonstrate that younger baseline age is associated with greater ΔHSDS and HV in these patients. Similar curves were observed with male and female patients for both ΔHSDS and first-year HV.

## Discussion

In this longitudinal study of GH treatment in patients with GHD, MPHD, TS, SGA, and ISS, HSDS improved over time. For patients with GHD, several variables were identified that correlated with growth response during the first and second years of GH treatment. HV at 4 months was the most significant predictor of ΔHSDS observed in the first 2 years of GH treatment. This observation that 4-month HV was such a strong predictor is a novel finding, since many studies do not consistently report growth this early in the treatment cycle. Additional factors that were influential in predicting HSDS outcomes were ranked in order of importance: younger baseline age > lower baseline HSDS > higher baseline BMI SDS > lower baseline IGF-I SDS.

For the GHD patient population, age and baseline HSDS were important determinants of the response to GH treatment, as previously demonstrated [18,20]. However, other reports have also indicated additional significant factors, such as birth weight SDS and GH dose [20]. The present results also indicated that higher baseline BMI was positively correlated with the growth response to GH for patients with GHD. Birth weight SDS and weight SDS were shown to be correlated with growth response to GH in the Pharmacia Kabi International Growth Study, suggesting that the heavier the child was, the greater the expected growth response to GH treatment [20]. The impact of BMI in this study might reflect, at least in part, the importance of nutrition for optimization of outcomes in patients receiving GH [1,33].

In general, the results from this analysis are consistent with previously published results for specific patient populations. A prior prediction study in patients with TS indicated that first-year growth response to GH was significantly influenced by weekly GH dose, chronological age, HSDS, body weight SDS, number of GH injections per week, and adjunctive oxandrolone treatment [26]. Predictors of the growth response over a longer duration of treatment (2-4 years) included HV during previous years, weekly GH dose, weight SDS, age, and oxandrolone treatment [26]. In SGA patients, results from one study found that first-year growth response to GH treatment was the most important predictor of second-year growth response [21]. Other variables that were significantly correlated with the growth response to GH included GH dose, weight and age at the start of treatment, and midparental HSDS [21]. Studies in the ISS patient population have identified additional factors as predictors of longer-term responses to GH, including baseline HSDS, GH dose, weight at the start of treatment, and first-year growth response [13,14]. It is important to recognize that this category may be the most heterogenous, with growth failure being a consequence of many different etiologies.

Specific results from other studies that are consistent with the present analysis, include the lack of gender effect on response to GH treatment. Analysis of results from the Pfizer Kabi International Growth Study database found no significant gender-related differences in effects of GH in HV or HSDS over 2 or 3 years of treatment [34]. In 8,018 patients with ISS in the National Cooperative Growth Study there was no significant effect of gender on first-year HV or first-year change from baseline in height SDS [35]. In a recent report, a large cohort of male and female patients with GHD, MPHD, TS, SGA, NS, and ISS from the ANSWER Program registry was used to assess gender-related differences in ΔHSDS following 2 years of GH treatment. Results demonstrated increased ΔHSDS in all patients, however, clinically relevant gender differences were not observed [36]. The importance of early timing for initiation of treatment from the present analysis is also consistent with previous findings. A National Registry of Growth Hormone Treatment report in the Netherlands that included 342 patients (diagnosis of GHD or a maximal GH response during provocation tests of less than 11 ng/mL) indicated that initiation of treatment before puberty resulted in a change from baseline in HSDS of 0.71 vs 0.58 for those who initiated treatment after puberty [19]. Results from the French registry of 2,852 patients with idiopathic GHD also indicated that prepubertal initiation of GH treatment was associated with significantly greater adult height gain [37]. Although in this study it is not known what proportion of patients across the different diagnostic categories may have been in puberty, the mean baseline chronological and bone ages are consistent with the majority of patients being prepubertal, and this likely lessens the impact of puberty on the observed growth responses.

The different correlations between baseline age, HSDS, BMI SDS, and IGF-I SDS, with growth response over 2 years of treatment with GH, carry implications for clinical practice. The correlation of baseline age with ΔHSDS and HV in the patients with GHD further support the initiation of GH at as young an age as possible to promote optimal growth. This concept is supported by results from another study that demonstrated a relationship between baseline age and first-year HV for patients with GHD, MPHD, and TS [15]. Several consensus statements endorse the use of GH treatment as soon as a diagnosis is made or growth failure is demonstrated for patients from several diagnostic categories [38-41]. The inverse relationship observed between baseline IGF-I and the two-year change in HSDS is consistent with an increased sensitivity to the effects of GH in patients who have a greater degree of GHD. In this non-interventional observational study, serum IGF-I was measured at a number of commercial laboratories reflecting routine clinical practice. IGF-I SDS was calculated using one formula which provided consistency to the analysis. This is also reflected in the finding that mean baseline IGF-I SDS in both the GHD and MPHD populations was lower than that observed in non-GHD patients. The positive correlation observed between baseline BMI SDS and ΔHSDS may emphasize the importance of nutrition in patients with growth failure [33,40]. An abnormally low BMI in pediatric patients may be a sign of malnutrition, which can also be associated with growth disturbances. In the end, the role of baseline age, HSDS, BMI SDS, and IGF-I SDS in the response of individual patients to GH therapy should all be considered for optimal management of short stature or growth failure.

## Conclusion

The present results from a large patient cohort enrolled in the ANSWER Program registry demonstrate gradual increases in ΔHSDS over time for all diagnostic categories. For patients with GHD, greater HV at 4 months is the most significant predictor of ΔHSDS over the first 2 years of GH treatment, while gender did not have any influence.

## Competing interests

PAL is a consultant for Abbott and Novo Nordisk, and has received clinical study support from Abbott, Novo Nordisk, Eli Lilly, Pfizer, and Ipsen. JR is a consultant for Novo Nordisk and Eli Lilly, and has received clinical study support from Novo Nordisk, Eli Lilly, and Pfizer. JG, RG, and NK are employees of Novo Nordisk.

## Authors' contributions

All authors contributed equally to this work and were involved in determining the study concept and design as well as providing data analysis and interpretation. RG and JG provided access to the registry data. NK performed the regression analysis. At all stages, the authors discussed the results and implications of the data and commented on the manuscript. All authors read and approved the final manuscript."
